# Transcriptional regulator ArcA mediates expression of oligopeptide transport systems both directly and indirectly in *Shewanella oneidensis*

**DOI:** 10.1038/s41598-019-50201-4

**Published:** 2019-09-25

**Authors:** Huihui Liang, Yinting Mao, Yijuan Sun, Haichun Gao

**Affiliations:** 1 Institute of Microbiology, College of Life Sciences, Zhejiang University, Hangzhou, Zhejiang 310058 China; 2 Research Center of Siyuan Natural Pharmacy and Biotoxicology, College of Life Sciences, Zhejiang University, Hangzhou, Zhejiang 310058 China

**Keywords:** Bacteriology, Bacterial genes

## Abstract

In γ-proteobacterial species, such as *Escherichia coli*, the Arc (anoxic redox control) two-component system plays a major role in mediating the metabolic transition from aerobiosis to anaerobiosis, and thus is crucial for anaerobic growth but dispensable for aerobic growth. In *Shewanella oneidensis*, a bacterium renowned for respiratory versatility, Arc (*So*Arc) primarily affects aerobic growth. To date, how this occurs has remained largely unknown although the growth defect resulting from the loss of DNA-binding response regulator *So*ArcA is tryptone-dependent. In this study, we demonstrated that the growth defect is in part linked to utilization of oligopeptides and di-tripeptides, and peptide uptake but not peptide degradation is significantly affected by the *So*ArcA loss. A systematic characterization of major small peptide uptake systems manifests that ABC peptide transporter Sap and four proton-dependent oligopeptide transporters (POTs) are responsible for transport of oligopeptides and di-tripeptides respectively. Among them, Sap and DtpA (one of POTs) are responsive to the *SoarcA* mutation but only *dtpA* is under the direct control of *So*ArcA. We further showed that both Sap and DtpA, when overproduced, improve growth of the *SoarcA* mutant. While the data firmly establish a link between transport of oligopeptides and di-tripeptides and the *SoarcA* mutation, other yet-unidentified factors are implicated in the growth defect resulting from the *So*ArcA loss.

## Introduction

Two-component systems (TCSs) are employed by prokaryotes to respond to constantly changing environmental conditions. In *Escherichia coli*, the Arc (anoxic redox control) system (*Ec*Arc) regulates gene expression in response to redox conditions^1,2^. *Ec*Arc consists of a transmembrane sensor kinase *Ec*ArcB and a DNA binding response regulator *Ec*ArcA^1^. Under anaerobic or microaerobic respiratory conditions, *Ec*ArcB undergoes autophosphorylation by sensing the redox state of quinone pool, eventually resulting in phosphorylated *Ec*ArcA (*Ec*ArcA-P) at the 54^th^ Asp residue through a phospho-relay mechanism^3,4^. *Ec*ArcA-P functions primarily as a global repressor of aerobic metabolic pathways, the tricarboxylic acid (TCA) cycle in particular. As a result, the *Ec*Arc system is crucial for anaerobic growth but dispensable for aerobic growth in *E. coli*^5,6^. Besides, as a global regulatory system, *Ec*Arc is implicated in diverse biological processes with a regulon consisting of hundreds of target genes^7,8^.

*Shewanella*, a group of facultative Gram-negative anaerobes renowned for their remarkable respiratory abilities, have become a research model for bacterial physiology^9^. As respiration is the predominant means for energy production in these bacteria, global regulators mediating metabolic transition in response to the availability of different electron acceptors (EAs) have been investigated for decades and many surprising observations have been made, especially in *S. oneidensis*, which is the most intensively studied representative of the genus.

The Arc system in *S. oneidensis* (*So*Arc) is atypical as the role of the sensor kinase is fulfilled by two proteins, ArcS (or ArcB1) and HptA^10–12^. Unlike *Ec*ArcB, which senses changes in the quinone composition, ArcS unlikely detects quinol species because it does not have the redox-sensitive cysteine residues of *Ec*ArcB^1,2^. Instead, ArcS has a periplasmic CaChe domain, which may function to sense yet-unknown extracellular signals^11^. This difference probably underpins the distinct influences of the two Arc systems on growth: *Ec*Arc is of the highest importance for micro-aerobic and anaerobic growth whereas *So*Arc plays a critical role in aerobic growth^13^. Despite this, activation of the *So*Arc system is similar to its *E. coli* counterpart through phospho-relay and 15-bp DNA-binding motifs for Arc proteins are highly conserved^6,11,13–15^. Interestingly, *E. coli* and *S. oneidensis* ArcA regulons differ from each other substantially; only 6 out of at least 50 members are shared^13^. Given that these two bacteria are rather close phylogenetically and there are more than 2,000 genes in common in *E. coli* and *S. oneidensis* genomes^1,13^, the difference is surprising. As a result, it is conceivable that the observed growth defect is not due to interference with expression of genes encoding established members of the *Ec*ArcA regulon^16,17^.

Further analyses of the *SoarcA* null mutant reveal that the growth defect is substantial in rich media such as Lysogeny broth (LB) but diminished in minimal media^13,16,18^. The growth defect re-emerges with addition of tryptone, suggesting that the *SoarcA* mutation may compromise the efficiency of oligopeptide metabolism^18^. Moreover, the *So*ArcA loss also results in a severely impaired cell envelope through yet-unknown mechanisms^19^. Given that *So*Arc is atypical and implicated in many biological processes clearly different from those revealed from bacteria hosting a canonical one such as *E. coli*, this regulatory system can serve as an ideal model to study respiration control for environmental microorganisms. The goal of this study was to unravel the mechanisms for the growth defect of the *SoarcA* mutant. We established that the major systems for oligopeptide (ATP peptide transporter Sap) and di-tripeptide (proton-dependent oligopeptide transporters, POTs) transport are subject to *So*ArcA regulation. Although these transporting systems are clearly implicated in promotion of aerobic growth on short peptides, only one of di-tripeptide transporter is under the direct control of *So*ArcA. As the growth defect of the *SoarcA* mutant could not be fully corrected by manipulated production of either system, we propose that the *SoarcA* mutation impairs growth through multi-fold complex impacts in physiology.

## Results

### The *arcA* mutation compromises peptide utilization in *S. oneidensis*

**Figure 1:**
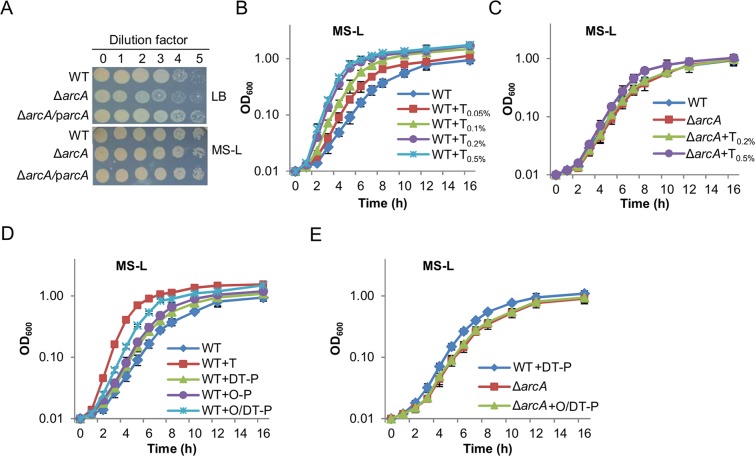
Comparative analysis of growth of the wild-type (WT) and the *arcA* mutant of *S. oneidensis*. (**A**) Growth on LB plates. Cultures of indicated strains prepared to contain approximately 10^9^ cfu/ml were regarded as the undiluted (dilution factor, 0), which were subjected to 10-fold series dilution. Five microliters of each dilution was dropped on indicated agar plates. Results were recorded after incubation of 18 h and 30 h for LB and MS-L respectively. Successful complementation by expressing a copy of the *arcA* gene *in trans* (ArcA) has been performed and reported repeatedly before. Data for cultures grown in liquid LB were shown in Fig. S1A. (**B**,**C**) Growth of WT and ∆*arcA* in MS-L without and with tryptone (T). Concentrations of tryptone were given in subscript. (**D**) Growth of WT in MS-L containing 0.5% oligopeptide (O-P) mixture, di-tripeptide (DT-P) mixture, or both (O/DT-P, refer to Table 1 for O-P and DT-P information). (**E**) Growth of ∆*arcA* in MS-L without or with 0.5% O/DT-P. Experiments were performed independently at least 5 times, and data were presented as the average and error bars representing standard errors.

Our preliminary analysis had demonstrated that the growth defect of the *S. oneidensis arcA* mutant in complex medium LB is dependent on tryptone^18^. As the *SoarcA* mutant cultivated in mineral medium MS with lactate as the carbon source (dubbed as MS-L throughout this study) is not defective with respect to aerobic growth (Figs 1A, S1A), this study was initiated by systematically assessing effects of tryptone on growth of the wild-type and ∆*SoarcA* strains in MS-L with tryptone addition. Clearly, growth of the wild-type increased with tryptone when its concentrations were no more than 0.2%, and became less sensitive to further augment to 0.5% in tryptone amounts (Fig. 1B). By generation times for 2 h during the exponential phase (Table S1), there was a 1.5-fold difference in growth rates between cells grown with 0.25% tryptone and without. In contrast, the ∆*SoarcA* strain was not responsive to the treatment with tryptone at 0.2% or lower, and grew slightly faster with 0.5% tryptone (Fig. 1C). These data verify that the *SoarcA* mutation compromises the growth- promoting effect of tryptone. Therefore, we reasoned that utilization of oligopeptides and/or amino acids is impaired by the mutation because tryptone is abundant in such nutrients, a result of incomplete enzymatic hydrolysis of casein.

To test this, impacts of tryptone, casamino acids, and an amino acid mixture on growth of the wild-type and ∆*SoarcA* strains were compared. In contrast to tryptone, neither casamino acids nor the amino acid mixture could elicit a significant difference in growth rates of both strains (Fig. S1B,C). This observation rules out the possibility that amino acids are an important factor responsible for the growth defect of the ∆*SoarcA* strain. Subsequently, we tested effects of oligopeptides (O-P, peptides of 4 and more residues, Table 1) of different lengths on growth of the wild-type and ∆*SoarcA* strains. Additions of a variety of oligopeptides only modestly improved growth of the wild-type (compared to that in MS-L) but showed no detectable effects on growth of the ∆*SoarcA* strain (Fig. 1D,E). Similar results were obtained with a mixture of di-tripeptides (DT-P) (Table 1). When both O-P and DT-P (O/DT-P) were present, growth of the wild-type was further accelerated whereas the ∆*SoarcA* strain remained unaffected. These data therefore suggest that the *SoarcA* mutant is defective in utilization of short peptides.

**Table 1:** Oligopeptide and di-tripeptide mixtures used in this study^a^.

Mixture	Sequence
**Oligopeptides**	
>5	RVYIHPFHL, KKLVFFA, YGGFLK, GRGDSP
5-	FLEEV, EHIPA, YGGWL, SFLLR
4-	FMRF, RGES, SFLR, SIGA, TDEV, NHAM
**Di-tripeptides**	
Tri-	GPR, Glutathione, GAY, GFR, RGD, VDR, YDS
Di-	AE, GG, GE, GQ, PE, KL, CI, YT, MD

^a^Concentrations of all peptides: 100 µg/ml.

### The ∆*SoarcA* strain is heavily defective in growing on O/DT-P

**Figure 2:**
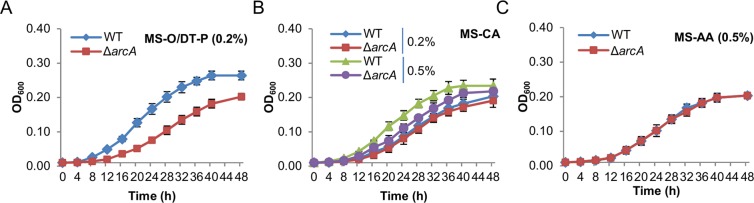
Defects of the *S. oneidensis arcA* mutant in aerobic growth are associated with peptide metabolism. (**A**) Growth of the wild-type (WT) and ∆*arcA* strains in MS with the peptide mixture (O/DT-P, 0.2%). (**B**) Growth the wild-type and ∆*arcA* strains in MS with casamino acids (CA, 0.2% and 0.5%). (**C**) Growth of the the wild-type and ∆*arcA* strains MS with the amino acid mixture (AA, 0.5%). More data for growth of WT and ∆*arcA* on these carbon sources were given in Fig. S2. Experiments were performed independently at least 5 times, and data were presented as the average and error bars representing standard errors.

Growth monitored above was in fact mainly supported by lactate, the carbon source in MS-L, which interferes with the contribution of the peptide mixtures under test. As an attempt to show the growth defect of the *SoarcA* mutant more clearly, growth on O/DT-P, casamino acids, and an amino acid mixture as carbon sources was assessed (Fig. 2A, 2B, 2C). Both the wild-type and ∆*SoarcA* strains were able to grow in MS with each of these carbon sources at concentrations up to 0.5% (Fig. S2). The amino acid mixture at all testing concentrations supported these two strains to grow comparably (Fig. 2C), manifesting that the *SoarcA* mutation does not have a significant impact on utilization of amino acids. In contrast, O/DT-P at all testing concentrations were not only more effective than the amino acid mixture in supporting growth, but more importantly, distinguished from the wild-type from the *SoarcA* mutant with respect to growth rate (Fig. 2A). Clearly, the difference in growth rates between them was substantial (Table S1). In the case of casamino acids, the growth difference between the two strains was negligible, noticeable, and significant with 0.1%, 0.2%, and 0.5% respectively (Fig. 2B). This further supports the impact of the *SoarcA* mutation in utilization of O/DT-P because small peptides make up ∼6% of casamino acids (250–500 (molecular weight), ∼6%, BD Bacto^TM^).

### Peptide digestion is not significantly affected by the *SoarcA* mutation

**Figure 3:**
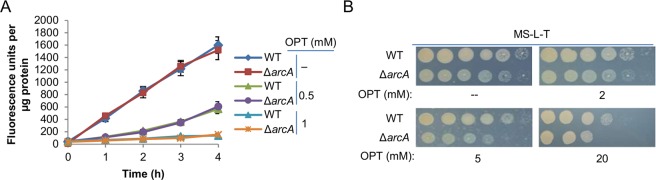
Contribution of peptide digestion to the growth defect of the *S. oneidensis arcA* mutant is negligible. (**A**) Metallaminopeptidase activity in cell extracts. An aliquot of cell extracts (of indicated strains grown to the mid-log phase in MS-L containing tryptone (MS-L-T)) was mixed with fluorescent peptide-AMC substrate. In parallel, a second aliquot was mixed with the substrate and OPT, a metalloprotease inhibitor. Fluorescence emission due to proteolytic cleavage of the substrate was measured in a microplate reader. (**B**) OPT susceptibility assay as performed the same as described in Fig. 1A on MS-L-T plates. In both panels, experiments were performed at least three times, with representative results or the average with error bars representing standard errors.

Utilization of peptides by the cell depends on two distinct steps, import from the outside and digestion into amino acids. To evaluate the contribution of peptide digestion to the growth difference between the wild-type and ∆*SoarcA* strains, we measured activity of metalloaminopeptidases, which are predominant in short peptide hydrolysis^20^. Both the wild-type and ∆*SoarcA* strains were grown in MS-L with tryptone to the mid-log phase and cells were collected by centrifugation. The pelleted cells were resuspended and disrupted by sonication with external cooling on ice, and after removal of cellular debris by centrifugation, cell extracts were assayed for aminopeptidase activity using fluorescent peptide-AMC (7-amido-4-methylcoumarin) substrates^21^. As shown in Fig. 3A, the amount of hydrolyzed peptide of the cell extracts from both the wild-type and ∆*SoarcA* strains linearly increased with time. The difference in peptidase activity between two strains, by normalized total protein amounts of each sample, was statistically insignificant. To verify that the hydrolysis is mainly attributed to metalloaminopeptidases, activities in the wild-type and ∆*SoarcA* cell extracts were assayed in the presence of 1,10-phenanthroline (OPT), a metal chelator functioning as unspecific and potent metalloprotease inhibitor^21^. Clearly, OPT inhibited the hydrolysis of peptide-AMC strongly; at 1 mM the maximum inhibition was achieved. In addition, we examined the effect of serineproteases on peptide hydrolysis. In the presence of 1 mM AEBSF (4-(2-aminoethlyl)benzenesulfony fluride), a broad spectrum and irreversible inhibitor for serineproteases^22^, the amount of fluorescence in the wild-type cell extracts was also affected (Fig. S3), suggesting a role of serineproteases in peptide digestion. However, given that the impact of AEBSF was substantially weaker than that of OPT, it is apparent that metalloaminopeptidases are a major contributing factor for peptide digestion. Importantly, the data obtained from the wild-type and ∆*SoarcA* strains were similar. Additionally, we performed the droplet assay to assess susceptibilities of the wild-type and ∆*SoarcA* strains to OPT and found that they were similar on MS-L plates containing tryptone (Fig. 3B). These data thus rule out the possibility that peptide hydrolysis is critically responsible for the growth defect of the ∆*SoarcA* strain.

### Selectivity of Sap and POTs in peptide transport

In bacteria, oligopeptide transport is predominantly mediated by transporters of three families: the proton-dependent oligopeptide transporter (POT), the ATP-binding cassette (ABC) peptide transporter, and the electrochemical potential-driven oligopeptide transporter (OPT)^23,24^. In the *S. oneidensis* genome, genes encoding POT and ABC peptide transporter system but not OPT are present^25^. For confirmation, the *S. oneidensis* proteome was screened for homologues of transporters in these three families whose oligopeptide transport activity has been established, including ABC peptide transporter systems of *E. coli* (OppABCDF)^26^ and *Pseudomonas aeruginosa* (DppABCDF)^27^, as well as a variety of POTs and OPTs typified by *E. coli* DtpA (formerly YdgR or TppB) and DtpB (formerly YhiP)^28,29^, and *Shewanella pealeana* Spea_3867 (Spe1)^23^. BLASTp screening revealed an ABC peptide transporter system, SapABCDF, and four POTs, SO_0002, SO_1277, SO_1505, and SO_3195 (Table 2). Among them, SO_0002 and SO_1277 have been proven to be di-tripeptide transporters biochemically^30,31^, and therefore are named as DtpA and DtpB respectively.

**Table 2:** *S. oneidensis* homologues of oligopeptide transporters verified in other bacteria.

Protein	Size (a.a.)	Verified Transporters (E-value)	Predicted function	
SapA (SO_1805)	541	*Pa*DppA (1e-130)	*Ec*OppA(3e-26)	ABC-type peptide uptake system
SapB (SO_1804)	343	*Pa*DppB(5e-81)	*Ec*OppB(4e-36)	
SapC (SO_1803)	296	*Pa*DppC(3e-78)	*Ec*OppC(5e-31)	
SapD (SO_1802)	335	*Pa*DppD(2e-73)	*Ec*OppD(1e-39)	
SapF (SO_1801)	261	*Pa*DppF(7e-72)	*Ec*OppF(2e-33)	
DtpA (SO_0002)	516	*Ec*DtpA(3e-29)	*Ec*DtpB(1e-18)	proton-coupled oligopeptide transporters
DtpB (SO_1277)	516	*Ec*DtpA(2e-112)	*Ec*DtpB (4e-113)	
SO_1505	514	*Ec*DtpA(4e,05)	*Ec*DtpB (>1)	
SO_3195	500	*Ec*DtpA(2e-65)	*Ec*DtpB(1e-59)	

**Figure 4:**
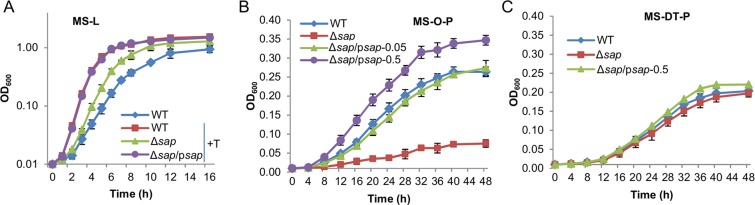
Characterization of Sap systems in *S. oneidensis*. (**A**) Growth of ∆*sap* and its complemented strains in MS-L or MS-L-T. Expression of *sap* on a plasmid (p*sap*) was driven by IPTG-inducible promoter P*tac*. With IPTG ranging from 0.05 to 0.5 mM, results were similar, with data from 0.5 mM being shown. Growth of the ∆*sap* strain in MS-L, similar to that of WT, was not shown for clarity. (**B**) Growth of ∆*sap* and its complemented strains in MS-O-P. (**C**) Growth of ∆*sap* and its complemented strains in MS-DT-P. In both B and C, numbers represent IPTG concentrations used for induction. Experiments were performed independently at least 5 times, and data were presented as the average and error bars representing standard errors.

To determine selectivity of Sap and POT peptide transport, we constructed deletion strains for each of them. While all four POT genes are transcribed from single-gene operons, a single operon, *sapABCDF*, encodes the ABC peptide transporter system. In MS-L containing tryptone, ∆*sap* (a strain lacking the entire *sap* operon) mutants showed a significant defect in growth, implicating that Sap plays a major role in transporting short peptides contained in tryptone (Fig. 4A). For complementation, we manipulated expression of the *sap* operon by using IPTG-inducible promoter P*tac*^32^. Defects of ∆*sap* in growth in tryptone-containing MS-L were corrected by expressing the missing *sap* genes *in trans* with IPTG ranging from 0.05 to 0.5 mM (Fig. 4A). According to previous calibration of the P*tac* promoter within pHGE, 0.5 mM IPTG confers the activity of the P*tac* promoter approximately 2000 Miller units^33–35^, more than 6 times of that of the *sap* promoter in the wild-type (refer to Fig. 6B). Therefore, these data, in addition to validate the correlation between the mutation and phenotypes, also indicate that the Sap peptide transporter produced in the wild-type is sufficient for importing short peptides contained in tryptone to support full growth on lactate as the carbon source. The selectivity of Sap was then investigated with O-P and DT-P as the carbon source. While the ∆*sap* strain grew only slightly slower than the wild-type on DT-P, it could barely do so on O-P (Fig. 4B,C). Expectedly, growth of the ∆*sap* strain on O-P was restored by expressing the *sap* operon in the presence of 0.05 mM IPTG. Moreover, we found that growth on O-P, unlike that in the tryptone-containing MS-L, further increased with IPTG concentrations (up to 0.5 mM), manifesting enhanced O-P uptake by overproduced Sap (Fig. 4B). On the contrary, Sap in overabundance had little effect on growth of the ∆*sap* strain on DT-P (Fig. 4C). These data indicate that the Sap system is primarily for uptake of peptides with 4 amino acid residues or larger.

**Figure 5:**
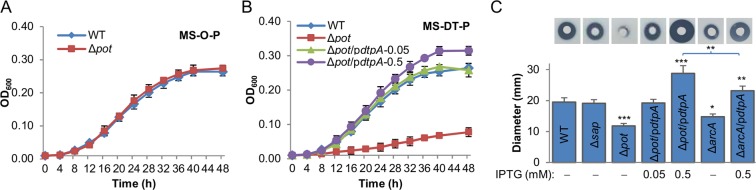
Characterization of POT systems in *S. oneidensis*. (**A**) Growth of the wild-type and ∆*pot* in MS-O-P. (**B**) Growth of the wild-type and ∆*pot* strains in MS-DT-P. Expression of *dtpA* on a plasmid (p*dtpA*) was driven by IPTG-inducible promoter P*tac*. Numbers represent IPTG concentrations used for induction. (**C**) Alafosfolin susceptibility determined by disk diffusion assay. Ten µl of 2 mg/ml alafosfolin was added to filter paper discs (8 mm) on cultures of indicated strains. Plates were incubated at 30 °C for 24 h. Asterisks not associated with a bracket represent statistically significant difference compared to the wild-type value (*P < 0.05; **P < 0.01; ***P < 0.001). Asterisks associated with the bracket represent the difference between the two linked strains. Experiments were performed independently at least 5 times, and data were presented as the average and error bars representing standard errors.

In the case of POTs, depletion of each POT alone did not significantly affect growth of the wild-type in tryptone-containing MS-L (Fig. S4). Although this observation implies that POTs are not critical in growth improvement by tryptone when lactate is used as the carbon source, it may be a result of functional overlapping among the four putative POTs. To test this, a strain (∆*pot*) devoid of all four POT genes was constructed and characterized with peptides as the carbon source. Compared to the wild-type, this strain displayed normal and substantially impaired growth on O-P and DT-P as the carbon source, respectively (Fig. 5A,B). The dependence of DT-P import on POTs was further verified by assessing the susceptibility of the ∆*pot* strain to alafosfolin, a di-peptide analog antibiotic that is a good substrate for POTs^28^: the ∆*pot* strain exhibited substantially elevated resistance whereas the ∆*sap* strain was almost unaffected (Fig. 5C). For complementation, we manipulated expression of the *dtpA* gene by using IPTG-inducible promoter P*tac*. Effects of *dtpA* expression on growth of the POT-deficient mutant on di-tripeptides were evident: significantly improved with 0.01 mM IPTG and fully restored to the wild-type level with 0.05 mM (Fig. 5B). DtpA in overproduction (0.5 mM IPTG) was able to further enhance growth on di-tripeptides and increased susceptibility of the ∆*pot* strain to alafosfolin (Fig. 5B,C). These data clearly manifest that POTs dictate transport of di-tripeptides, and that these POTs, at least some of them, can functionally complement one another.

### Peptide transport has a role in explaining the ∆*SoarcA* growth defect

**Figure 6:**
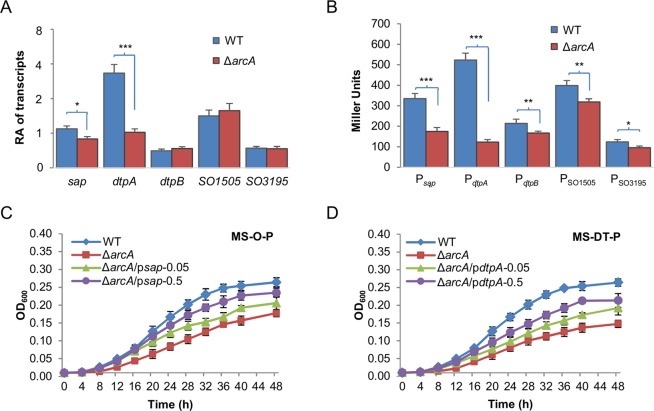
Peptide transport is partially accountable for the *S. oneidensis* ∆*arcA* growth defect. (**A**) Expression levels revealed by qRT-PCR assay. The wild-type and ∆*arcA* strains grown to the mid-log phase in MS containing 0.5% tryptone were used for the assay. The averaged values for each gene under test were normalized to that of the *recA* gene, giving to relative abundance (RA) of transcripts. Asterisks indicate statistically significant difference (*P < 0.05; **P < 0.01; ***P < 0.001). (**B**) Promoter activity assay. Indicated promoters were used to drive expression of the full-length *E. coli lacZ* gene within an integrative system. β-galactosidase activities in the mid-log phase cells were determined and presented as Miller Units. (**C,D**) Growth of the wild-type and ∆*arcA* strains in MS-O-P or MS-DT-P. Overexpression of *sap* and *dtpA* was achieved as described above. Numbers represent IPTG concentrations used for induction. Experiments were performed independently at least 5 times, and data were presented as the average and error bars representing standard errors.

To determine whether these peptide transporters are implicated in the growth defect of the ∆*SoarcA* strain, we compared expression of their coding genes in the wild-type and the mutant with qRT-PCR. Cells of both strains grown with the short-peptide mixture as the carbon source to the mid-log phase were used. Analysis of transcripts of these genes revealed that the *sap* and *dtpA* genes displayed significantly reduced transcription in the ∆*SoarcA* strain, approximately by 30% and 3-fold respectively, while the remaining three genes were transcribed comparably in these two strains (Fig. 6A). We then utilized an integrative *lacZ* reporter system for confirmation^36^. DNA fragments of ∼400 bp upstream of the coding sequences for these operons, which are sufficiently long to cover entire promoters for most of operons, were amplified and cloned into the reporter vector pHGEI01. The resulting vectors, verified by sequencing, were introduced into relevant *S. oneidensis* strains for integration and subsequent removal of the antibiotic marker^37^. From similarly prepared cells, we surprisingly found that the differences in expression of the *sap* and *dtpA* genes were enhanced, and *dtpB* and *SO_1505* were also modestly down-regulated in the mutant (Fig. 6B). These data indicate that at least *sap* and *dtpA* are subject to positive regulation of *So*ArcA, although it is clear that more investigations are needed to address the difference in data revealed by these two methods.

To assess whether the down-regulated expression of *sap* or *dtpA* is responsible for the growth difference between the wild-type and ∆*SoarcA* strains on O-P and DT-P, we monitored growth of the ∆*SoarcA* strain with *sap* and *dtpA* expressed at different levels. Consistent with compromised production of Sap and POTs, the *SoarcA* mutant exhibited significantly impaired growth on O-P and DT-P (Fig. 6C,D). Importantly, the growth difference between the wild-type and ∆*SoarcA* strains on O-P and DT-P decreased with forced expression of *sap* and *dtpA* respectively. In the presence of 0.05 mM IPTG, these transporters significantly improved growth of the *SoarcA* mutant on the respective carbon source (Fig. 6C,D), contrasting undetectable effects on the growth of the wild-type (Fig. S5). These data support that the *SoarcA* mutant suffers from impairment in peptide import. In addition, we found that the *SoarcA* mutant with overproduced Sap and DtpA by 0.5 mM IPTG grew at further accelerated rates, but it still was inferior to the wild-type (Fig. 6C,D). For comparison, the same treatments enable ∆*sap* and ∆*pot* strains to grow faster than the wild-type (Figs 4B, 5B). Thus, while these data indicate that peptide transport has a role in determining growth of the *SoarcA* mutant, other factors are involved.

### *dtpA* is under the direct control of *So*ArcA in *S. oneidensis*

**Figure 7:**
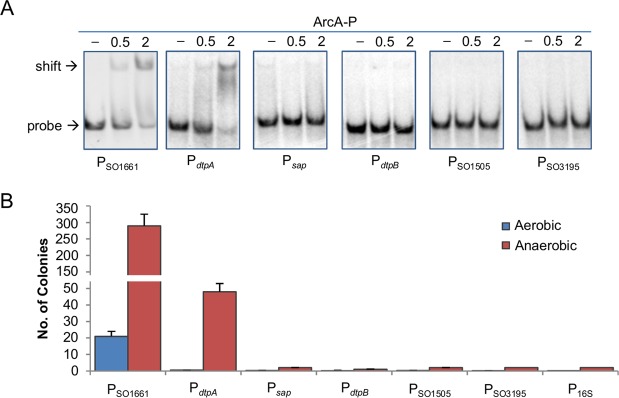
Only *dtpA* is under the direct control of ArcA in *S. oneidensis*. (**A**) EMSA assay. Experiments were performed in the presence of 0, 1, or 2 μM ArcA-P and 2–5 nM radiolabeled promoter DNA. 0.2 μg/μl poly(dI·dC) was used in all these binding reactions to block non-specific interactions. The phosphorylation of the ArcA protein was done with carbamoyl phosphate. The images shown were cropped from different gels. (**B**) B1H assay. Experiments were carried out as described in Materials and Methods. Colonies for the positive interactions were from M9 salt agar plates containing 25 mg/ml chloramphenicol and 12.5 mg/ml tetracycline with or without 3-amino-1,2,4-triazole (3-AT) after 24 h incubation and confirmed by streaking colonies on plates containing both 3-AT and streptomycin (12.5 mg/ml). P_SO1661_ and P_16S_ were used as positive and negative controls. Experiments were performed independently at least 5 times, with representative results or the average with error bars representing standard errors.

The promoter region of *dtpA* (encoding one of POTs) but not the *sap* operon has been predicted to have an *So*ArcA-binding site (GGTAAATAGATGTAA; *E. coli* and *S. oneidensis* consensus, GTTAATTAAATGTTA)^13,14^. However, the confidence for this site is rather low, based on its weight value (6.5) obtained from genome screening. To test whether these genes are under the direct control of *So*ArcA, electrophoretic motility shift assay (EMSA) was used with the recombinant *S. oneidensis* His_6_-tagged *So*ArcA protein produced in *E. coli* and purified as described before^13,14^. As phosphorylation of *So*ArcA is required for its specific binding, only *So*ArcA (*So*ArcA-P) phosphorylated by carbamoyl phosphate was used. The DNA fragments, approximately 300 bp in length centered by the predicted *So*ArcA-binding motif (if available) of the genes to be tested, were amplified with ^33^P end-labeled primers. Among the tested PCR sequences in EMSA with phosphorylated *So*ArcA, *dtpA* showed *So*ArcA(-P) binding activity (Fig. 7A). In contrast, retardation of the sequences of *sap* and genes for other two POTs was not observed.

Interactions *in vivo* between *So*ArcA and *dtpA* and *sap* promoter region sequences were then tested with bacterial one-hybrid (B1H) analysis, which has proven to be a robust technique applicable to a wide variety of different transcriptional factor families and has been frequently used in *S. oneidensis*^33,38,39^. This is enabled by the fact that *E. coli arcB* expression restores the wild-type phenotype of an *arcS*/*hptA* deletion in *S. oneidensis* and that, vice versa, upon ectopic production ArcS/HptA can functionally complement the loss of *EcarcB* in *E. coli*^10–12^. In the B1H system, vectors containing ‘bait’ (DNA) and ‘target’ (DNA-binding regulator) are co-transformed into reporter cells and positive interaction between them allows growth in the presence of 3-amino-1,2,4-triazole (3-AT). When cultured under aerobic conditions, growth was detected only from the positive control pair *So*ArcA/P_*SO_1661*_ (Promoter region of SO_1661)^13,14^ with 20 colonies on average (Fig. 6B). Given that similar studies with other regulators/cognate DNAs usually generate ∼300 colonies^33,39^, we reasoned that this low efficacy is likely due to that i) *Ec*ArcA proteins may compete for the target DNA and ii) most of *So*ArcA proteins are not phosphorylated under test conditions. To test the first possibility, the *EcarcA* gene was knocked out from the reporter strain and the resulting mutant was verified by genetic complementation with respect to increased sensitivity to many dyes such as toluidine blue and crystal violet^40,41^ (Fig. S6A). However, with this strain the B1H assay gave out similar results (Fig. S6B), indicating that the interference from *Ec*ArcA is negligible. To improve phosphorylation, we cultivated cells under fumarate-respiring conditions. In the absence of oxygen, more than 200 colonies were obtained from the reporter strains with P_*SO_1661*_/*So*ArcA, either with or without the *EcarcA* gene (Figs 6B, S6B). Importantly, the reporter strains containing P_*dtpA*_/*So*ArcA was able to form 50 or so colonies on 3-AT plates whereas P_*sap*_/*So*ArcA failed to support growth on 3-AT. These data, collectively, indicate that *So*ArcA directly regulates transcription of *dtpA* but not genes for other peptide transporters in *S. oneidensis*.

## Discussion

The Arc system is among the most intensely investigated regulatory systems in γ-proteobacteria and its physiological function(s) known to date is largely derived from studies on the *E. coli* paradigm. However, as a TCS, the Arc system is rather unusual. First, the Arc system is only known to exist in four γ-proteobacteria families: Alteromonaceae, Enterobacteraceae, Vibrionaceae, and Pasterurellaceae, although its components, OmpR-family regulators and hybrid sensor histidine kinases, are common in bacteria^1^. Second, unlike most of TCSs, the components of all Arc systems, including both the typical (ArcB-ArcA) and the atypical (without a full length ArcB), are never encoded by genes in a single operon, not even in proximity in most cases^42^. Third, the atypical Arc system, as in *S. oneidensis*, is in fact quite common among the Alteromonads^1^.

Although regarded to be a global regulator with pleiotropic impacts in physiology, the canonical Arc system functions primarily as a global repressor of aerobic metabolic pathways^2,43^. However, this may be one side of the regulatory effects about Arc systems. There are only several overlaps in the ArcA-controlled regulons of *E. coli* and *S. oneidensis*, indicating that the physiological function of *S. oneidensis* ArcA is substantially different from that of the *E. coli* counterpart^13^. The best example is growth under aerobic conditions. In contrast to the *E. coli* paradigm, which appears to have a rather limited role during aerobic growth, the *So*Arc system is critical^6,13,44^. However, the growth phenotype resulting from the *So*ArcA loss is conditional, unlike the defect in cell envelope, another major defect which is independent of growth parameters tested.

The dependence of the growth phenotype on tryptone indicates that it is associated with nutrients, oligopeptides in particular^18^. Following this lead, in this study we examined roles of peptide transporters and peptidases in *S. oneidensis*. Peptidases, including both metalloaminopeptidases and serineproteases, are dispensable for the growth defect resulting from the *So*ArcA loss, ruling out the possibility that peptide hydrolysis is critically involved. On the contrary, oligopeptide transport is implicated. ABC peptide transporter consisting of SapABCDF and four POTs are primarily responsible for uptake of oligopeptides and di-tripeptides respectively. By using both qRT-PCR and *lacZ*-reporters, we concluded that expression of *dtpA* (encoding one of POTs) and *sap* is significantly down-regulated in the absence of *So*ArcA. Importantly, both DtpA and Sap, when manipulatedly produced, are able to improve growth of the *SoarcA* mutant, supporting a link between the growth defect resulting from the *So*ArcA loss and peptide transport. Despite this, it is clear that there are other factors implicated in the growth defect of the *SoarcA* mutant.

We also presented evidence from both *in vitro* and *in vivo* experiments to show that transcription of *dtpA* but not the *sap* operon is directly mediated by *So*ArcA. The interaction between *So*ArcA and the *dtpA* promoter region appears weak, likely due to low conservation of its *So*ArcA-binding site as suggested before^13,14^. In *S. oneidensis* and *E. coli*, it has been demonstrated that phosphorylation is essential for biological activity of ArcA and ArcA can be phosphorylated under anaerobic as well as aerobic conditions^8,10–12,15,19,45^. However, studies from us and others have argued that *So*ArcA proteins exist mostly in the phosphorylated form when cells grow aerobically^11,13,15^, contrasting the scenario observed in *E. coli* because the histidine kinase domain of its *Ec*ArcB is activated under anaerobic conditions^45,46^. Results of the B1H analysis performed in *E. coli* certainly support this proposal. In *E. coli* cells grown aerobically, positive interactions were detected only between *So*ArcA and DNA fragments imbedded with a highly conserved binding motif (SO_1661). Under oxygen-free conditions, more *Ec*ArcB became activated, which in turn phosphorylated more *So*ArcA. As a result, *So*ArcA exhibited stronger binding to the SO_1661 promoter, and more importantly, it was able to interact with the *dtpA* promoter region, validating that *So*ArcA directly regulates transcription of the *dtpA* gene *in vivo*.

During the investigation, we noted a significant difference in expression levels of genes for peptide transporters obtained from qRT-PCR and *lacZ*-reporters. With the *lacZ*-reporters, all five operons were found to be down-regulated in the mutant whereas qRT-PCR only differentiated *dtpA* and *sap*. We speculate that this may be due to compromised translation efficacy in the absence of the *So*Arc system. Translation is carried out by ribosome and there is a linear relation between the ribosome mass fraction and the growth rate^47^. This understanding resonates with our previous findings that the *SoarcA* mutation concertedly down-regulates the majority of components in protein synthetic machinery^13,18^. In addition, a remarkable number of translation-associated proteins, such as translation initiation and elongation factors and tRNA synthases, are present in less amounts in the ∆*SoarcA* strain than the wild-type with respect to transcripts and proteins. On the contrary, the *So*ArcA loss has no effect on expression of most of the transcription machinery members. We are working to test this notion.

The Arc system of *E. coli* was initially recognized to confer resistance to dyes such as toluidine blue O and methylene blue, photosensitizers that facilitate generation of reactive oxygen species in the presence of light^40,48^. However, the underlying mechanism for this phenotype remains largely elusive, implying that it may be unexpectedly complex^41,49^. In parallel, we previously showed that the cell envelope of *S. oneidensis* is impaired in the absence of the Arc system. A whole-genome screening with dual-effect transposon constructs, which are able to both inactivate genes by interruption and overexpress genes after the insertion site, fails to identify suppressors for the phenotype^19,50^. The failure suggests that the observed envelope defect, the same as the growth defect, unlikely due to altered expression of single gene/operon, is a result of multi-fold complex impacts in physiology. We continue our effort to identify the important proteins for both the growth and envelope defects as they are the key to better understand the physiological significance of this atypical Arc system.

## Materials and Methods

### Bacterial strains, plasmids, and culture conditions

A list of all bacterial strains and plasmids used in this study was given in Table 3. Information for primers used in this study was given in Table S2. Chemicals are from Sigma-Aldrich Co. unless otherwise noted. *E. coli* and *S. oneidensis* strains under aerobic conditions were grown in Lysogeny Broth (LB, Difco, Detroit, MI) medium, which was modified to contain tryptone (10 g/L), yeast extract (5 g/L), and NaCl (5 g/L), at 37 °C and 30 °C for genetic manipulation. When needed, the growth medium was supplemented with chemicals at the following concentrations: 2,6-diaminopimelic acid (DAP), 0.3 mM; ampicillin sodium, 50 µg/ml; kanamycin sulfate, 50 µg/ml; and gentamycin sulfate; 15 µg/ml.

**Table 3:** Strains and plasmids used in this study.

Strain or plasmid	Description	Reference or source
***E. coli*** **strains**		
DH5α	Host for cloning	Lab stock
BL21	Protein expression host	GE Healthcare
WM3064	Δ*dapA*, donor strain for conjugation	W. Metcalf, UIUC
XL1-Blue	Reporter strain for B1H assay	Stratagene
EC4401	Δ*arcA* derived from XL1-Blue	This study
***S. oneidensis*** **strains**		
MR-1	Wild type	Lab stock
HG3988	∆*arcA* derived from MR-1	^19^
HG0002	∆*dtpA* derived from MR-1	This study
HG1277	∆*dtpB* derived from MR-1	This study
HG1505	∆*SO1505* derived from MR-1	This study
HG1801-5	∆*sap* derived from MR-1	This study
HG3195	∆*SO3195* derived from MR-1	This study
POT	∆*pot* derived from MR-1, all *pot* genes removed	This study
**Plasmid**		
pHGM01	Ap^R^, Gm^R^, suicide vector for mutant construction	^52^
pHGEI01	Km^r^, integrative *lacZ* reporter vector	^42^
pHGE-Ptac	Km^r^, IPTG-inducible expression vector	^39^
pBBR-Cre	Sp^r^, helper plasmid for antibiotic cassette removal	^14^
pHG101-arcA	Complement vector carrying *arcA*	^26^
pBXcmT	B1H bait vector	^43^
pTRG	B1H target vector	^43^
pDEST17-ArcA	Expressing vector for *arcA*	^19^
pKD46	Red recombinase expression vector	^53^
pHGEI01-P*sap*	P*sap*-*lacZ* fusion within pHGEI01	This study
pHGEI01-P*dtpA*	P*dtpA*-*lacZ* fusion within pHGEI01	This study
pHGEI01-P*dtpB*	P*dtpB*-*lacZ* fusion within pHGEI01	This study
pHGEI01-P*SO1505*	P*SO1505*-*lacZ* fusion within pHGEI01	This study
pHGEI01-P*SO3195*	P*SO3195*-*lacZ* fusion within pHGEI01	This study
pHGE-Ptac-sap	P*tac*-*sap* within pHGE-Ptac	This study
pHGE-Ptac-dtpA	P*tac*-*dtpA* within pHGE-Ptac	This study
pHG101-EcarcA	Complement vector carrying *E. coli arcA*	This study

For all other purposes, cells of *S. oneidensis* strains grew in MS mineral medium [KCl, 1.34 mM; NaH_2_PO_4_, 5 mM; Na_2_SO_4_, 0.7 mM; NaCl, 52 mM; piperazine-*N*,*N* = -bis(2-ethanesulfonic acid) (PIPES), 3 mM; NH_4_Cl, 28 mM; MgSO_4_, 1 mM; CaCl_2_, 0.27 mM; FeCl_2_, 3.6 µM, pH 7.0]^51^. When supplemented with sodium lactate (30 mM by default), or each of the following (0.5% by default): tryptone, casamino acids, an oligopeptide mixture, a di-tripeptide mixture, an oligo- and di-tri-peptide mixture, and an amino acid mixture (Table 1), media were named as MS-L, MS-T, MS-CA, MS-O-P, MS-DT-P, MS-O/DT-P, and MS-AA, respectively. The amino acid mixture was composed of 500 µg/ml of each alanine, cysteine, glycine, histidine, aspartic acid, glutamic acid, phenylalanine, asparagine, glutamine, methionine, leucine, isoleucine, proline, serine, threonine, lysine, and valine, 50 µg/ml of tryptophan and tyrosine. The MS basal medium was sterilized by in an autoclave, and required supplements sterilized by filtration were added before usage. Fresh medium was inoculated with overnight cultures grown from a single colony by 1:100 dilution, and growth was determined by recording the optical density of cultures at 600 nm (OD_600_). For anaerobic growth, mid-log phase aerobic cultures were pelletted by centrifugation, purged with nitrogen, suspended in fresh medium containing 20 mM fumarate as the electron acceptor prepared anaerobically to an OD_600_ of ∼0.02.

### Mutant construction and complementation

In-frame deletion strains derived from *E. coli* MG1655 and *S. oneidensis* MR-1 were constructed by the Red recombination deletion method and the *att*-based Fusion PCR method, respectively^52,53^. In the case of *S. oneidensis*, two fragments flanking the target gene were amplified by PCR with outside primer containing *attB* and the gene specific sequence and inside primer containing the linker and the gene specific sequence, which were joined by the second round of PCR with two outside primers. The fusion fragments were introduced into plasmid pHGM01 by using Gateway BP clonase II enzyme mix (Invitrogen) according to the manufacturer’s instruction, resulting in mutagenesis vectors, which were maintained in *E. coli* DAP auxotroph WM3064. The vectors were subsequently transferred into relevant *S. oneidensis* strains via conjugation. Integration of the mutagenesis constructs into the chromosome was selected by resistance to gentamycin and confirmed by PCR. Verified transconjugants were grown in LB broth in the absence of NaCl and plated on LB containing 10% sucrose. Gentamycin-sensitive and sucrose-resistant colonies were screened by PCR for deletion of the target gene. Mutants were verified by sequencing the mutated regions.

Growth of *S. oneidensis* mutation strains generated in this study was measured by recording the optical density at 600 nm (OD_600_) values in triplicate with the wild-type as the control in MS- based media as mentioned above^51^. For genetic complementation of the mutants and inducible gene expression, genes of interest generated by PCR were placed under the control of Isopropyl β-D-1-thiogalactoside (IPTG)-inducible promoter P*tac* within pHGE-P*tac*^32^. After verification by sequencing, the resultant vectors in *E. coli* DAP auxotroph WM3064 were transferred into the relevant strains via conjugation.

### Peptidase activity assays

Fluorescence peptidase assays were performed using L-Leu-AMC substrate^21^. Reaction mixtures contained 20 mM Tris-Cl (pH 7.5), 10 μM L-Leu-AMC, and 150 μl of extracts of cells grown in MS-L-T to the mid-log phase. The peptidase activity was monitored at 460 nm with a Synergy 2 Pro200 Multi-Detection (Microplate Reader (Tecan) by detection of 7-amino-4-methylcoumarin (MCA) released from the carboxyl terminus of N-terminally blocked peptides. Negative controls (no enzyme) were run in triplicate to account for thermal degradation of substrates as the background. One unit of protease activity was defined as the amount of enzyme required to release 1 μmol of MCA per min per mg protein. Protein concentration was determined with a bicinchoninic acid assay kit with bovine serum albumin as a standard according to the manufacturer’s instructions (Pierce Chemical).

### Droplet assays

Droplet assays were employed to evaluate growth inhibition on plates^54^. Cells of the mid-log phase (were collected by centrifugation and adjusted to 10^9^ cfu/ml (colony forming unit), which was set as the undiluted (dilution factor 0). Ten-fold series dilutions were prepared with fresh medium indicated. Five microliters of each dilution was dropped onto LB or MS-L-T plates containing required agents as indicated in the figure. The plates were incubated for 24 h or longer before being read. All experiments were conducted at least three times.

### Disc diffusion assays

Disc diffusion assays were done similarly to those done previously^39^. Briefly, overnight cultures were diluted into LB and grown to the mid-log phase. One hundred microliters of cultures was spread onto an agar plate containing required chemicals. Paper discs (diameter, 8 mm) containing 10 μl of 2 mg/ml alafosfolin were placed on top of the agar. The plates were incubated at 30 °C for 16 h prior to analysis. The diameters of the zones of clearing (zones of inhibition, in millimeters) were measured. All assays were done in triplicate using independent cultures, and the resulting zones of inhibition were averaged.

### Analysis of gene expression

For qRT-PCR, *S. oneidensis* cells were grown in MS containing 0.5% tryptone with the required additives to the mid-log phase and collected by centrifugation, and RNA extraction was performed using the RNeasy minikit (Qiagen) as described before^55^. RNA was quantified by using a NanoVue spectrophotometer (GE Healthcare). The analysis was carried out with an ABI7300 96-well qRT-PCR system (Applied Biosystems). The expression of each gene was determined from three replicates in a single real-time qRT-PCR experiment. The Cycle threshold (*C*_*T*_) values for each gene of interest were averaged and normalized against the *C*_*T*_ value of the *recA* gene, whose abundance is relatively constant during the log phase. Relative abundance (RA) of each gene was presented.

Activity of target promoters was assessed using a single-copy integrative *lacZ* reporter system as described previously^36^. Briefly, fragments containing the sequence upstream of the target operon were amplified, cloned into the reporter vector pHGEI01, and verified by sequencing. The resultant vector in *E. coli* WM3064 was then transferred by conjugation into relevant *S. oneidensis* strains, in which it integrated into the chromosome and the antibiotic marker was removed subsequently^37^. Cells grown to the mid-log phase under conditions specified in the text and/or figure legends were collected and β-galactosidase activity was determined by monitoring color development at 420 nm using a Synergy 2 Pro200 Multi-Detection Microplate Reader (Tecan) presented as Miller units.

### Electrophoretic motility shift assay (EMSA)

Expression and purification of His-tagged *S. oneidensis* ArcA has been described before^13,19^. Phosphorylation of purified *So*ArcA was performed in buffer containing 100 mM Tris/HCl (pH 7.0), 10 mM MgCl_2_, 125 mM KCl, 50 mM dilithium carbamoyl phosphate for 60 minutes at room temperature. The probes used for EMSA were prepared by PCR with ^33^P end-labeled primers^13^. The binding reaction was performed with ∼25-50 fmol (∼2-5 nM) labeled probes and various amounts of protein in 12 µl binding buffer containing 100 mM Tris/HCl (pH 7.4), 20 mM KCl, 10 mM MgCl_2_, 2 mM DTT, 0.2 μg/μl poly(dI·dC), and 10% glycerol at 15 °C for 60 minutes and resolved on pre-run 4.8% polyacrylamide native gels. Band shifts were visualized by autoradiography.

### Bacterial one-hybrid (B1H) assay

B1H system was used to investigate DNA-protein interaction *in vivo* in *E. coli* cells^38^. Briefly, plasmid constructs were created by cloning the bait ‘DNA’ and target ‘the *SoarcA* gene’ in the pBXcmT and pTRG vectors and verified by sequencing. The resultant plasmids were used to co-transform BacterioMatch II Validation Reporter Competent Cells on M9 salt agar plates containing 25 mg/ml chloramphenicol and 12.5 mg/ml tetracycline with or without 3-amino-1,2,4-triazole (3-AT). DNA fragments of ∼300 bp for SO_1661 and the 16s rRNA gene promoters (P_SO1661_ and P_*16s*_ respectively) were used as positive and negative controls. The plates were incubated for 24 h and then moved to room temperature for an additional 16 h (the colonies indicating positive interaction usually appeared between 18 and 24 h). For the assay under anaerobic conditions, plates were incubated in an anaerobic glovebox (Coy Manufacturing Co.) until the colonies were fully developed. The positive interactions were confirmed by streaking colonies on plates containing both 3-AT and streptomycin (12.5 mg/ml).

### Other analyses

Homologues of proteins of interest were identified via a BLASTp search of the NCBI’s nonredundant protein database, using the amino acid sequence as the query. Experimental values were subjected to statistical analyses and presented as means ± standard error of the mean (SEM). Student’s *t*-test was performed for pairwise comparisons of groups.

## Supplementary information

ALl supplemental materials
