# Supplementary material for: Transcriptional regulator ArcA mediates expression of oligopeptide transport systems both directly and indirectly in Shewanella oneidensis
Source: Sci Rep. 2019 Sep 25;9:13839. doi: 10.1038/s41598-019-50201-4 (PMC6761289; doi:10.1038/s41598-019-50201-4)
Supplement: Supplementary file 1 — ALl supplemental materials [file 41598_2019_50201_MOESM1_ESM.pdf]

Supplemental Materials of

Transcriptional regulator ArcA mediates expression of oligopeptide transport systems both directly and indirectly in *Shewanella oneidensis*

Huihui Liang[1],† Yinting Mao[1],† Yijuan Sun[1,2], and Haichun Gao[1,2]*

[1]Institute of Microbiology and [2]Research Center of Siyuan Natural Pharmacy and Biotoxicology, College of Life Sciences, Zhejiang University, Hangzhou, Zhejiang, 310058, China

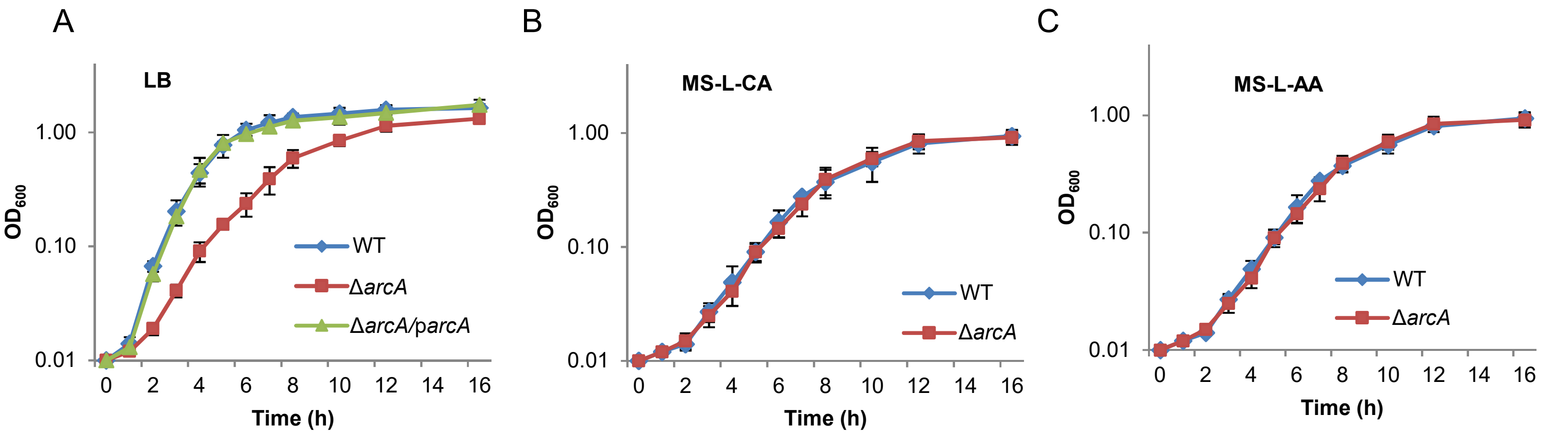

FIG S1 Growth of the wild-type (WT) and Δ*arcA* strains in LB or defined medium MS with lactate as the carbon source (MS-L). Growth was recorded by measuring OD_{600} values of cultures. Complementation, shown as Δ*arcA/*p*arcA,* was carried out by expressing a copy of the *arcA* gene *in trans*. CA, casamine acids, 0.5%; AA, a mixture of amino acids, 0.5%. Experiments were performed independently at least 3 times, and data were presented as the average and error bars representing standard errors.

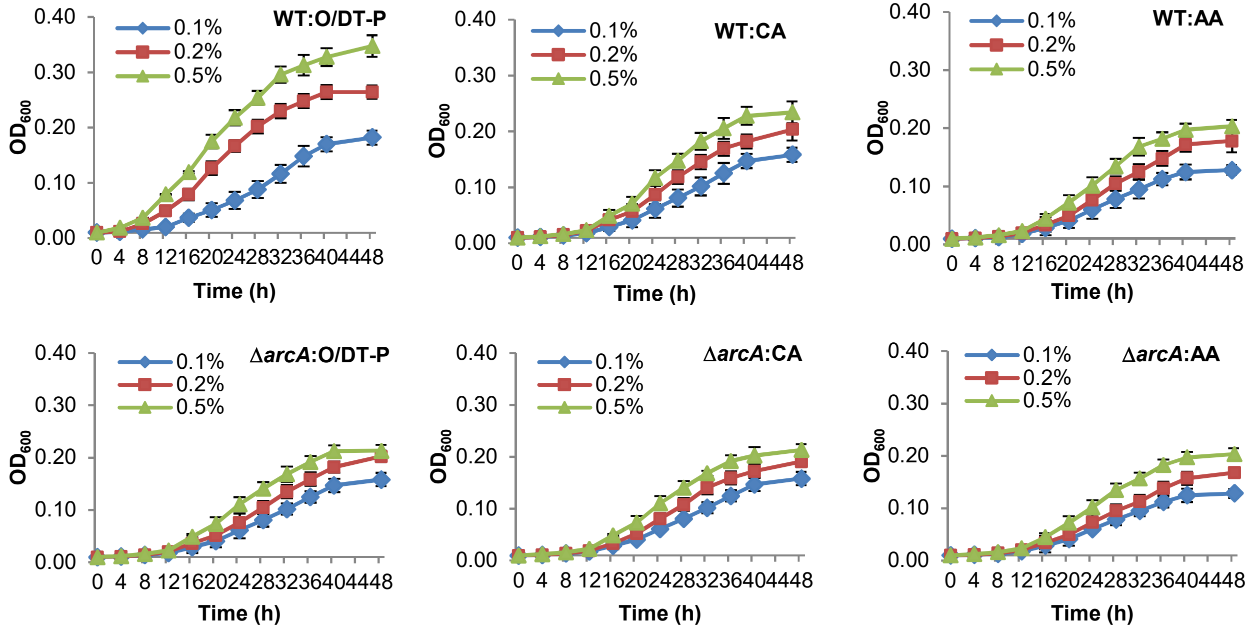

FIG S2 Growth of the wild-type (WT) and Δ*arcA* strains in MS with the oligopeptide and di-tripeptide (O/DT-P) mixture, casamino acids (CA), the amino acid mixture (AA) at indicated concentrations. Growth was recorded by measuring OD_{600} values of cultures. Experiments were performed independently at least 3 times, and data were presented as the average and error bars representing standard errors.

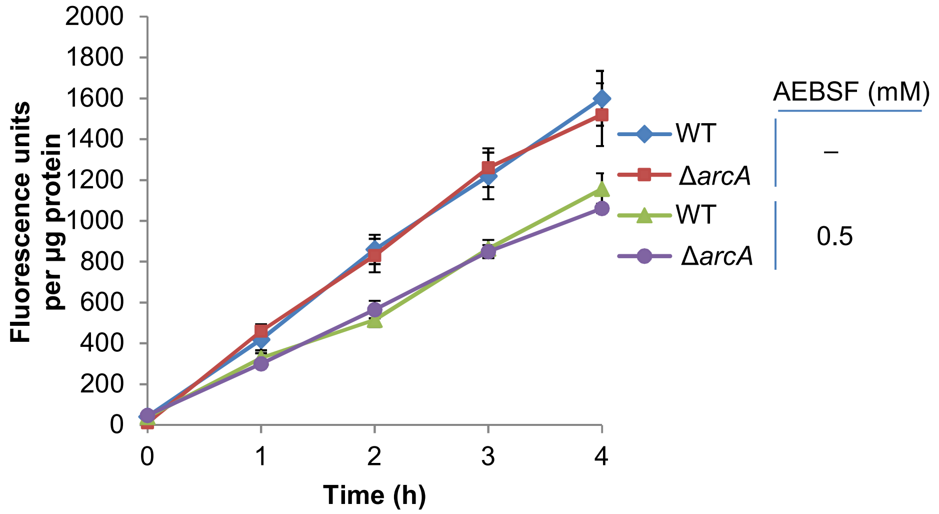

FIG S3 Serinepeptidase activity in *S. oneidensis* strains. An aliquot of cell extracts (of indicated strains grown to the mid-log phase in MS-L containing 0.5% tryptone) was mixed with fluorescent peptide-AMC substrate. In parallel, An second aliquot was mixed with the substrate and AEBSF, a serineprotease inhibitor. Fluorescence emission due to proteolytic cleavage of the substrate was measured in a microplate reader. Experiments were performed independently at least 3 times, and data were presented as the average and error bars representing standard errors.

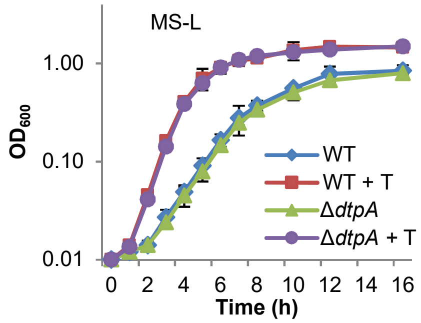

FIG S4 Characterization of POT systems in *S. oneidensis*. Growth of Δ*dtpA* in MS-L without or with 0.5% tryptone (T). The same results were obtained from Δ*dtpB*, Δ*SO_1505*, and Δ*SO_3195*. Experiments were performed independently at least 5 times, and data were presented as the average and error bars representing standard errors.

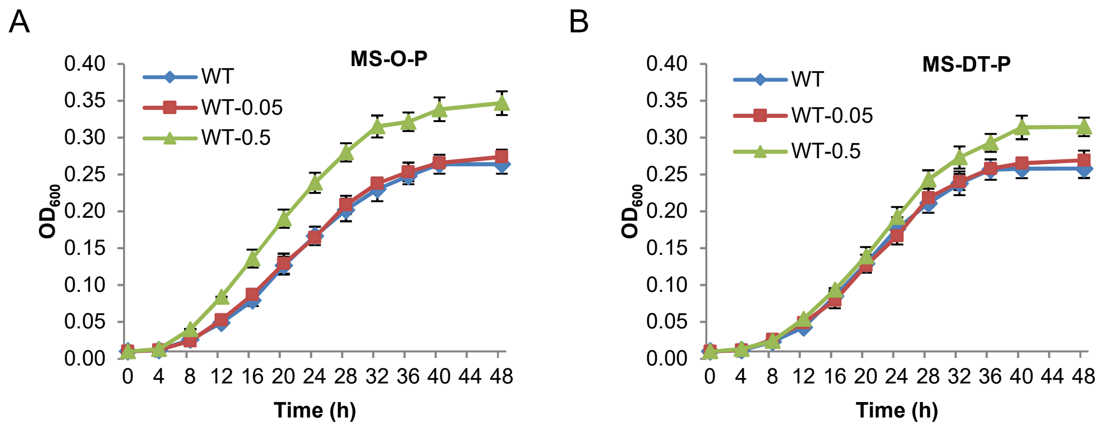

FIG S5 Impacts of additional expression of peptide import systems on the wild-type. (A) Growth of the wild-type in MS containing 0.5% O-P. (B) Growth of the wild-type in MS containing 0.5% DT-P. Expression of *sap* and *dtpA* was achieved as described above. Numbers represent IPTG concentrations used for induction. Experiments were performed independently at least 5 times, and data were presented as the average and error bars representing standard errors.

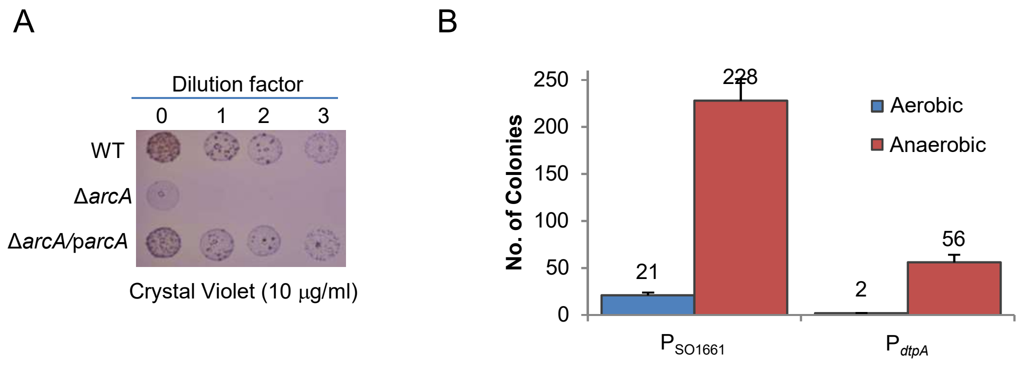

FIG S6 The presence of *E. coli* ArcA has little effect on the B1H assay. (A) Growth of ArcA-less B1H reporter strain on LB plates containing crystal Violet. Cultures of indicated strains were prepared as described in Fig. 1. Results were recorded after incubation of 24 h. Genetic complementation by expressing a copy of the *arcA* gene *in trans* was carried out with the P*tac* promoter in the presence of 0.1 mM IPTG. (B) B1H assay with the ArcA-less reporter strain as performed in Fig. 7B. Experiments were performed independently at least 5 times, with representative results or the average with error bars representing standard errors.

Table S1 Generation times of *S. oneidensis* strains (minutes)

Media / Strains	MS-L	MS-L + $T_{0.25\%}$	MS-L + $T_{0.5\%}$	MS-L + O/DT-P	MS-L + O-P	MS-L + DT-P	MS + O/DT-P	MS + O-P	MS + DT-P
WT	75	51	46	71	84	87	220	240	360
Δ*arcA*	78	75	69	104			355	330	370
Δ*sap*			85					>600	
Δ*pot*							220		>600
Δ*sap/*p*sap*			55					250	330
Δ*pot*/p*dtpA*									350
Δ*arcA*/p*sap*								280	
Δ*arcA*/p*dtpA*									320

TABLE S2 Primers used in this study

Primers	Primer sequences
**In-frame deletion**	
HG0002-M5O	GGGGACAAGTTTGTACAAAAAAGCAGGCTGCGTTGGATGCAGGAAAGTA
HG0002-M5I	GGTCCGGGTTCGCTATCTATACGATCAGCTATCCAACCGC
HG0002-M3I	ATAGATAGCGAACCCGGACCGATGAGCGTGACGGCATTCC
HG0002-M3O	GGGGACCACTTTGTACAAGAAAGCTGGGTGCCCGATTTTATCAATCGCC
HG1277-M5O	GGGGACAAGTTTGTACAAAAAAGCAGGCTTACCAGCCTTTAAGATAGTG
HG1277-M5I	GGTCCGGGTTCGCTATCTATTCATCAAAGCCAAGACGCTG
HG1277-M3I	ATAGATAGCGAACCCGGACCGTACTTAGGCGGCGTTGTGG
HG1277-M3O	GGGGACCACTTTGTACAAGAAAGCTGGGTGCCATTGGCCCCGGTGGTTT
HG1505-M5O	GGGGACAAGTTTGTACAAAAAAGCAGGCTTACCAAACCCGATTAGCACG
HG1505-M5I	GGTCCGGGTTCGCTATCTATGTGGAGCCAAAAATGGTTTC
HG1505-M3I	ATAGATAGCGAACCCGGACCGCGCGGCATGAGCCCTACAG
HG1505-M3O	GGGGACCACTTTGTACAAGAAAGCTGGGTTATGGCAGTTAAAGCAACTG
HG1801/5-M5O	GGGGACAAGTTTGTACAAAAAAGCAGGCTCCTTAACGAGCCCGCCTGTC
HG1801/5-M5I	GGTCCGGGTTCGCTATCTATAGGAAAACAGCACATCATCG
HG1801/5-M3I	ATAGATAGCGAACCCGGACCGTAGCGCGAGCACTCATGCT
HG1801/5-M3O	GGGGACCACTTTGTACAAGAAAGCTGGGTCTTCATGGCTAAAGGCATCG
HG3195-M5O	GGGGACAAGTTTGTACAAAAAAGCAGGCTCTAAAGCATAGCCGCTCGGT
HG3195-M5I	GGTCCGGGTTCGCTATCTATTACAGATCACCCACCATAGT
HG3195-M3I	ATAGATAGCGAACCCGGACCTGGTATTGCGATTACCGCAG
HG3195-M3O	GGGGACCACTTTGTACAAGAAAGCTGGGTGCTAGGTCTATTGGACTGGG
**Controlled expression**	
Sap-CEF	GGGAATTCATGAGTGTGCTAATAAGACGCCTTTGCCTA
Sap-CER	CGGGATCCGTGGTTGGAGCAGTTTAACGTTTGT
DtpA-CEF	GGGAATTCATGACTACACCTGTTGATGCGCC
DtpA-CER	CGGGATCCAGTTATCACTTATGTCGCTTGCCG
***lacZ* reporters**	
P*sap*-F	GGGAATTCCCCTTAACGAGCCCGCCTGTC
P*sap*-R	CGGGATCCCTGCAGTGCATCTCGCCCATGG
P*dtpA*-F	GGGAATTCGCACCTGCGCCGTGGGTGGAT
P*dtpA*-R	GGGAATTCCTCTGTTATCCTATTATAATTG
P*dtpB*-F	GGGAATTCCGTACCAGCCTTTAAGATAGTG
P*dtpB*-R	CGGGATCCTGAAAGCTTGCTTCCGTTATTG
P*SO1505*-F	CGGGATCCCCTACCAAACCCGATTAGCACG
P*SO1505*-R	CGGGATCCTTGTCCTTTCCTTTATTATTGT
P*SO3195*-F	CGGGATCCCCTCTAAAGCATAGCCGCTCGGT
P*SO3195*-R	CGGGATCCTTGAAGTTTCCACCTTAAGCTC
